# Supplementary figures and images for: A GBS-Based GWAS Analysis of Leaf and Stripe Rust Resistance in Diverse Pre-Breeding Germplasm of Bread Wheat (Triticum aestivum L.)
Source: Plants (Basel). 2022 Sep 10;11(18):2363. doi: 10.3390/plants11182363 (PMC9504680; doi:10.3390/plants11182363)

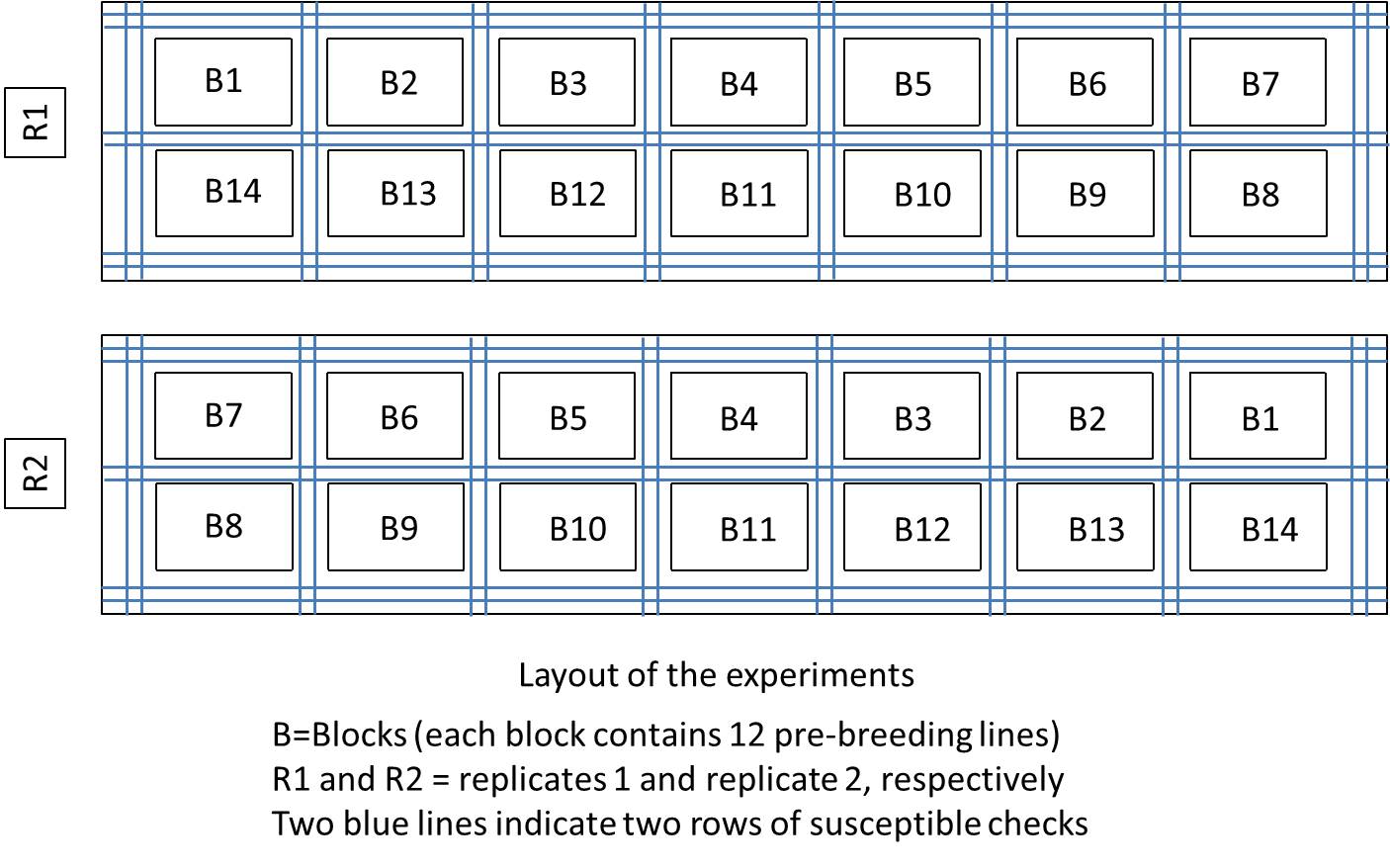

Supplement: Supplementary file 1 [file plants-11-02363-s001.zip › Fig S1.jpg]
